# Perceived barriers and attitudes of health care providers towards Provider-Initiated HIV Testing and Counseling in Mbeya region, southern highland zone of Tanzania

**DOI:** 10.4314/pamj.v8i1.71070

**Published:** 2011-03-03

**Authors:** A Kapologwe Ntuli, Julieth S Kabengula, Sia E Msuya

**Affiliations:** 1 Health Department, Mbeya City Council, Mbeya, Tanzania; 2 KCM College, Tumaini University, Moshi, Tanzania; 3 Muhimbili National Hospital, Dar es Salaam, Tanzania; 4 KCMC Hospital, Moshi, Tanzania

**Keywords:** Attitude, barriers, provider-initiated, testing and counseling, HIV, Tanzania

## Abstract

**Background:**

Provider-initiated testing and counseling (PITC) is a routine HIV testing and counseling, it encompases two strategies including; diagnostic HIV testing and HIV screening. In Tanzania PITC started in 2007, to date it is almost through out the country. This study aimed at assessing the perceived barriers and attitudes of health care providers towards PITC services.

**Methods:**

A cross sectional study was conducted for one month between April and May, 2010 in the goverment health care facilities of the Mbeya City Council. A multi-stage sampling technique was used to select both health facilities and health care providers.

**Results:**

A total of 402 (95%) subjects were interviewed. Their mean (± SD) age was 41 ± 9.5 years, where majority (65%) were females. All the participants reported to be aware about PITC services. However, about 35% of them had negative attitude towards PITC services. Various perceived barriers to effective PITC provision were reported, including; too many patients (57.7%) and inadequate space (46%) for PITC provision.

**Conclusion:**

Although PITC is an effective strategy for identification of unrecognized HIV infections, there is still missed opportunity which occurs at the health facilities, as some of health care providers had negative attitude and others faces various barriers in offering the PITC service.

## Background

Human Immunodeficiency Virus (HIV) and Acquired Immunodeficiency Syndrome (AIDS) is a global health threat and it continues to pose great public health challenges. It is estimated that, about 33.4 million people are living with HIV globally [1]. In the year 2008, approximately 2.7 million people were infected with HIV and 2 million died from AIDS [1]. Sub-Saharan Africa is the most affected area and currently it is estimated to have 70% of people living with HIV and contributing 68% of the new HIV infections globally [1]. HIV is a public health problem also in Tanzania with a prevalence of 5.7% among adults aged 15–49 years [2]. The prevalence however, varies substantially between regions and districts in the country. For example the prevalence of HIV was reported to be 15.7% at Kilolo District in Iringa region and 12.5% for Mbeya City Council which is twice of the reported national prevalence of 5.7%. Other regions like Kigoma reported a very low prevalence of 2% [2].

Despite the high prevalence of HIV and high number of new infections in sub-Saharan Africa (SSA), few people know their HIV status. Previous studies have reported that only 10% - 12% of people in sub-Saharan Africa know their HIV status, despite long term efforts to promote and support voluntary counseling and testing (VCT) for HIV [1]. In Tanzania it was reported that only about 15% of the people are aware of their HIV sero-status [3].

Since 2007, UNAIDS and WHO has recommended that countries with generalized HIV epidemics had to adopt a policy of provider initiated HIV counseling and testing (PITC) in clinical settings. The policy guidelines suggest that, HIV counseling and testing should be recommended by the health care provider as part of routine care to all patients or clients attending the facilities, regardless of whether the patient shows signs and symptoms of underlying HIV infection [2]. PITC encompasses two strategies in which health care providers are required to perform. These includes HIV counseling, diagnostic HIV testing, and HIV screening. The guideline also emphasize that, PITC should be voluntary and that the “Three C’s” – informed consent, counseling and confidentiality must be observed for both forms of PITC [2]. The current PITC policy is intended to complement the existing HIV VCT programs which rely on individuals to self-refer for testing [2-12].

In Tanzania, PITC was introduced and then scaled up since 2007. In Mbeya Region, the scale-up of PITC was started in late 2007. This has been achieved through training of health care providers facilitated by Ministry of Health and Social Welfare (MOHSW) in collaboration with nongovernmental organisations. Health care workers are thus important for the sucess of PITC services. Assessing the health care providers’ attitudes and perceived barriers towards PITC provision is critical in ensuring delivery of PITC services according to the WHO recommendation. But there is limited information on PITC since its introduction in 2007. This study therefore, aimed to assess perceived barriers and attitude towards PITC provision in the health facilities of Mbeya City Council, Tanzania.

## Methods

A cross-sectional study was conducted from April to May, 2010 among health care providers (nurses and clinicians), working in the government health facilities of Mbeya City Council, Southern Tanzania. Mbeya city council has a poulation of about 358,939 people, served with one refferal hospital, one regional hospital and ten government primary health care facilities (dispensaries and health centres). The council has a total of 720 health care workers, among them 510 are nurses and 210 are clinicians.

**Figure 1:**
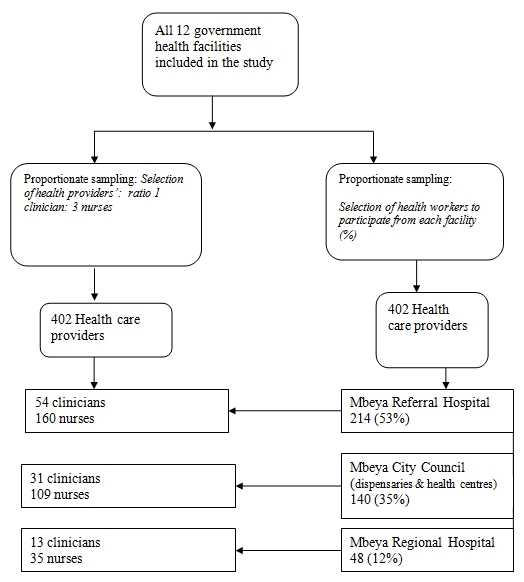
Sampling scheme used for the selection of participants

Study site involved all of the 12 government health facilities in Mbeya City Council. Proportionate to size sampling was used to select the participants. Fifty three percent of the participants were from Mbeya referral hospital, 35% were from primary health care facilities and 12% were from Mbeya regional hospital (Figure 1). Stratification of clinicians and nurses at a ratio of 1 clinician to 3 nurses was done at each facility. Simple random sampling was then used to select the participants from a list of names provided by health secretaries of the respective health care facilities. The minimum required sample size was 422 participants, however 402 (95%) health care providers agreed to participate.

Self administered structured questionnaire with both closed and open ended questions was used to collect information on socio demographic variables, PITC-knowledge, attitudes and barriers from the study participants. The language used was Swahili as it is understood by every Tanzanian. The principal investigator informed the selected health workers about the study and its aims and a signed consent was obtained for those agreeing to participate. A questionnaire was then given to the participants and they were required to fill and submit them to the investigator within 48 hours.

A total of ten questions, using four point Likert scale were used to assess attitude towards PITC. The questions asked on how health workers felt regarding different aspects of PITC (Table 1). Each question had a maximum score of 4 points giving the total score of 40 points. All the questions had equal weight and were grouped into; 1 = strongly disagree, 2 = disagree, 3 = agree and 4 = strongly agree. However when attitude was assessed for question number 8 (PITC should be considered to only clients with high risk), the scores were reversed, with strogly disagree scoring 4 points and strongly agree scoring 1 point.

Analysis of participants’ attitude was done after dichotomization of the four-point-likert scale questions, that is “strongly disagree” and “disagree” became “disagree” and “strongly agree” and “agree” became “agree”. Those who scored 30 points and above were categorized as having “positive attitude”, while those who scored below the cut-off point were categorized as having “negative attitude”. Scores were then used to categorize the respondent’s attitude into two major categories {Category I= Positive attitude, Category II= Negative attitude}.

Barriers to PITC were assessed using one open ended question. The answers were categorised into themes and coded. Coded data was then analysed in similar manner as closed ended responses.

Data collected were entered and analysed using Statistical Package for Social Science (SPSS) program version 17.0 (SPSS Inc., Chicago, IL, USA). Descrptive statistics were used to summarize the data.

The ethical approval for the study was obtained from the KCM-College research ethics comittee. Permission to conduct the study was also sought from the administrative authority of the respective study areas. Informed signed consent was obtained from all the participants. Both confidentiality and privacy issues were taken into account and only numbers were used instead of names.

## Results

A total of 402 (95%) health care workers participated in the study. Their mean age was 41 (SD ± 9.5) years. Most of the participants were females (65%), nurses by profession (76%), were ever married (79%), and had certificate or diploma (74%). Their years of practice varied from 4 months to 39 years with median years of practice of 9 years (IQR, 4-20 years) (Table 2).

All of the 402 health care providers reported to have heard about PITC. The common reported sources of information on PITC were; from a professional colleague (66.4%), followed by training on PITC (53.5%) and with 23.9% reported to have heard about PITC from conferences.

Almost two thirds of the health care providers had positive attitude towards PITC while 34.8% (n=140) demonstrated negative attitude (table 1). Among the ten attitude items tested, most of the respondents agreed to statements that were positive towards PITC service delivery. However, some responses showed negative attitude for example; 63.2% of the health workers (n=254) felt that PITC was too much involving and took too much time and 77.9% (n=313) were of the opinion that, PITC should be considered only for clients at high risk of having HIV infection, thus more focus should be to those who are symptomatic and those who are attending STI clinics. Predictors for positive attitude towards PITC were evaluated. None of the socio-demographic characteristics, years of practice, and health facility characteristics were statistically significant associated with attitude towards PITC.

**Figure 2:**
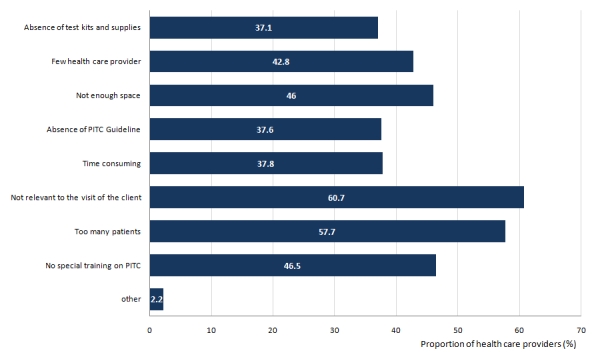
Barriers towards ProviderInitiated HIV Testing and Counseling (PITC) provision by health care providers (n = 402)

Perceived barriers to offer PITC services as reported by health providers are shown in figure 2. PITC not being relevant to the client visit was the most common reported barrier (61%), as health care providers thought that, not all patients were at risk of acquiring HIV infection. Other perceived barriers were too many patients at the clinics (57.7%), there were few health care providers (42.8%), lack of special training on PITC (46.5%) and absence of test kits (37.1%).

## Discussion

The health care workers are the cornerstone for implementation of PITC strategy. In addition, PITC helps people to learn their HIV status and be able to link to care, treatment and support services earlier. In this study, most of the health care providers (96%) reported feeling comfortable in offering PITC services to the clients visiting the health care facilities. Similar observation was reported at San Francisco General Hospital in USA in which, majority of health workers (84.2%) reported feeling very or mostly comfortable with consenting patients for HIV testing [4]. Despite feeling comfortable, about 78% of health care providers’ attitude was to recommend PITC to people at risk of having HIV infection as it is time consuming. This attitude is contrarily to the PITC principle of offering HIV counseling and testing to every patient or client attending the health facility for any reason, so as to detect early the asymptomatic HIV infection.

The attitude of risk assessment before offering PITC to the client was the most common barrier reported by health providers in our setting. In this study about 61% of the health providers reported that, discussing PITC service with a client is difficult especially when the patient is not symptomatic or has a complaint that is not related to the HIV/AIDS. These findings do not differ from results of the study done in USA on STD/HIV prevention practices among primary care clinicians which revealed that, many clinicians (63%) provided PITC services to patient they considered to be of high risk and most (83%) health workers were asking only specific HIV risk questions to patients who had a history of STD or had symptoms of STD. This attitude has serious implications for a place like Mbeya City Council, with high HIV prevalence (12.5%) [2-12]. Special efforts are urgently required to emphasize to these health care providers the importance of early detection of HIV infection among clients. For countries like Tanzania which has a generalized HIV epidemic, the need to give opportunity for every person visiting the health care setting to be counseled and tested cannot be over emphasized considering HIV care and treatment services are now readily available up to the primary health facilities.

Nearly all (95%) of the health providers had an attitude that, “effective PITC can reduce fear of client from testing”. This finding is similar to the results observed in a population based study done in Botswana on routine HIV testing; attitudes, practices and human rights concerns. Of 1,268 adults who participated in that study, 83% agreed that routine HIV testing will make it easier for people to get tested and gain timely access to antiretroviral therapy and in the long run it will help to break the stigma at community level [6]. PITC is widely accepted by the health care providers of Mbeya City Council, however some health facilities factors were barriers to PITC provision. Forty three of the health providers mentioned constraints in human resources and 58% mentioned having to attend too many patients as barriers to PITC provision in this study. According to the Ministry of Health and Social Welfare report of 2008; most government health facilities have a shortage of between 65% and 70% required health care providers [7]. It seems that the few health care providers available felt overwhelmed by an additional work load required to offer PITC. A report by Chair of Michelsen Institute (CMI) on human resources for health in Tanzania, supports the finding showing it is difficult to deliver quality health services against shortage of human resources with skills [8]. Both central and local government leaders need to have a concentrate strategy on how to address the problem of human resources for health.

Lack of motivation or incentives and poor working conditions (i.e. absence of PITC guidelines, HIV test kits and inadequate physical space) were other challenges in offering PITC in this setting. A study done in Kilimanjaro, Tanzania among health care workers showed that, motivation was among the important factor in health care performance [9].

Health care providers also pointed out that lack of training on PITC, too much time used in the testing process and that PITC strategy might not be relevant to the visit of asymptomatic clients as barriers. All these point to the fact that a different model of increasing the chance to detect unrecognized HIV infection in the population which is efficient and accepted by both health workers and community is required. One of the alternative might be home based HIV counseling and testing done by community health workers. This will help to reduce excessive patient load in the facilities.

Despite the importance of the information generated from this study, there were some limitations. The study was a cross-sectional in nature and relied on self-reports. The weakness of the reported information without observation is known. Secondly, the tool used in assesing attitudes to PITC has never been validated before, so it might be difficult to generalize the findings of this study to other settings.

## Conclusion

Although PITC is an effective strategy for identification for unrecognized HIV infections in Mbeya, Tanzania, there are some barriers for effective implementation. Negative attitude to PITC by a third of health providers, shortage of staff, excesive patient load and inadequate space are some of the barriers identified. Health managers need to to urgently address the human resource constrain as well as space and supply of consumables at the facilities. Further, studies are required to look at alternative models of PITC provision which are more efficient and acceptable by health care providers.

## Competing interests

The authors declare that there were no competing interests.

## Authors’ contribution

Ntuli Kapologwe contibuted to data collection, analysed the data and prepared the manuscript. Sia Msuya and Julieth Kabengula contributed to data collection, data analysis and reviwed the manuscript. All authors read and approved the final manuscript.

## Acknowledgements

We thank the the Tanzanian Ministry of Health and Social Welfare for funding this study. Authors wish to acknowledge health care providers, District Medical Officer, Medical officers in-charge of all health facilities which participated in this study and also the authorities of Mbeya City Council for their cooperation. Special thanks should go to Imani Mwasomola and Dinah Atinda for their support during data collection.

## Figures and Tables

**Table 1:** Scores of participants on each attitude towards PITC question (N= 402)

	**Agree^1^**	**Disagree^2^**
**Attitude variables**	**No. (%)**	**No. (%)**
PITC is important in the clinical practice	374 (93.0)	28 (7.0)
PITC can be offered without any problem	363 (90.3)	39 (9.7)
Health care providers are ready to offer PITC	359 (89.3)	43 (10.7)
PITC is much involving to health care providers	254 (63.2)	148 (36.8)
PITC offers more opportunity for HIV testing	354 (88.1)	48 (11.9)
There are more benefits of offering PITC than VCT	339 (84.3)	63 (15.7)
Client without any HIV symptoms still can be offered PITC	369 (91.8)	33 (8.2)
PITC should be considered to only with high risk group*	313 (77.9)	89 (22.1)
Feeling comfortable offering PITC services	385 (95.8)	17 (4.2)
Effective PITC can reduce fear of client from HIV testing	380 (94.5)	22 (5.5)

^1^Agree includes all who responded “strongly agree=4” and “agree=3 ”.^2^Disagree includes all who responded “disagree=2” and “strongly disagree=1.” *Score is different 1= strongly agree, 2= agree, 3= disagree, 4= strongly disagree. NB: One participant could respond more than one response

**Table 2:** Social-demographic characteristics of 402 healthcare workers in Mbeya City council, Tanzania

**Variable**	**Frequency (n)**	**Percent (%)**
**Sex**		
Female	260	64.7
Male	142	35.3
Age (years) Mean (SD*, Range)	**41 (9.5, 24-60)**	
Younger age ( ≤ 35 years)	303	75.4
Older age ( ≥36 years)	99	24.6
**Religion**		
Christian	344	85.6
Muslim	58	14.4
**Marital status**		
Never married	85	21.1
Ever married	317	78.9
**Highest qualification attained**		
Lower qualification	298	74.1
Higher qualification	104	25.9
**Profession**		
Nurse	304	75.6
Clinician	98	24.4
Years of practice: Median (IQR)**	**9 (4-20)**	
Short in service employees (≤ 10 years)	237	75.6
Long in service employees (≥ 11 years)	165	24.4

*SD = Standard Deviation, **IQR= Interquartile range
